# Cathepsin S as an early biomarker for cardiovascular disease in chronic kidney disease patients

**DOI:** 10.1590/2175-8239-JBN-2021-0135

**Published:** 2022-01-12

**Authors:** Satyendra Kumar Sonkar, Prashant Kumar Singh, Sharad Chandra, Gyanendra Kumar Sonkar, Vivek Bhosale, Sharad Sharma

**Affiliations:** 1 King George's Medical University, Medicine Department, Lucknow, India.; 2 King George's Medical University, Cardiology Department, Lucknow, India.; 3 King George's Medical University, Biochemistry Department, Lucknow, India.; 4 Central Drug Research Institute, Clinical & Experimental Medicine, Lucknow, India.

**Keywords:** Diabetes Mellitus, Hypertension, Echocardiography, Atherosclerosis, Arteriosclerosis, Diabetes Mellitus, Hipertensão, Ecocardiografia, Aterosclerose, Arteriosclerose

## Abstract

**Introduction:**

A high incidence of cardiovascular disease (CVD) events and premature mortality is observed in patients with chronic kidney disease (CKD). Thus, new biomarkers that may help predict the development of CVD in early stages of CKD are being investigated along with other traditional risk factors.

**Objective:**

To investigate cathepsin S as an early biomarker for CVD in patients with CKD.

**Methods:**

A total of 64 patients with CKD were included and classified into 2 groups: CKD patients with established CVD and CKD patients with non-established CVD. All patients were submitted to routine investigations including complete blood count, random blood sugar, glycated hemoglobin (HbA1c), serum electrolytes, urea, creatinine, total protein, total albumin, calcium total, phosphorous, uric acid, vitamin D, parathormone, lipid profile, liver function test, measurement of serum cathepsin S (Cat S), and 2D Echo of the heart.

**Results:**

The level of serum Cat S was increased in CKD patients with CVD (p <0.05) as well as in later stages of CKD (p <0.05). CVD was also more common in patients in early stage CKD. In early stages CKD, Cat S and CVD were positively correlated.

**Conclusion:**

These findings suggest that serum Cat S might be useful as an early biomarker for CVD in CKD patients.

## Resumo

**Introdução:**

Uma alta incidência de eventos de doença cardiovascular (DCV) e mortalidade prematura é observada em pacientes com doença renal crônica (DRC). Assim, novos biomarcadores que podem ajudar a prever o desenvolvimento de DCV nos estágios iniciais da DRC estão sendo investigados juntamente com outros fatores de risco tradicionais.

**Objetivo:**

Investigar a catepsina S como um biomarcador precoce para DCV em pacientes com DRC.

**Métodos:**

Um total de 64 pacientes com DRC foram incluídos e classificados em 2 grupos: pacientes com DRC com DCV estabelecida e pacientes com DRC com DCV não estabelecida. Todos os pacientes foram submetidos a investigações de rotina incluindo hemograma completo, glicemia aleatória, hemoglobina glicada (HbA1C), eletrólitos séricos, ureia, creatinina, proteína total, albumina total, cálcio total, fósforo, ácido úrico, vitamina D, paratormônio, perfil lipídico, teste de função hepática, medição da catepsina S sérica (Cat S), e Eco 2D do coração.

**Resultados:**

O nível de Cat S sérica esteve aumentado em pacientes com DRC com DCV (p <0,05), bem como em estágios posteriores da DRC (p <0,05). A DCV também foi mais comum em pacientes com DRC em estágio inicial. Em estágios iniciais da DRC, a Cat S e a DCV foram positivamente correlacionadas.

**Conclusão:**

Estes achados sugerem que a Cat S sérica pode ser útil como um biomarcador precoce para DCV em pacientes com DRC.

## Introduction

Chronic kidney disease (CKD) is a worldwide public health problem, with adverse outcomes of end-stage kidney failure^1^. There is a very high incidence of cardiovascular disease (CVD) events and premature mortality in patients with CKD^2^, with a sharp increase in risk as glomerular filtration rate (GFR) declines below 60 mL/min/1.72m^2^ 3. CVD is closely associated with CKD and shares many common risk factors. It is reported that about 50% of patients with CKD die from cardiovascular causes due to the acceleration in the development of atherosclerotic plaques^4^.

Cathepsins are inactive proenzymes that are activated by the proteolytic removal of the N-terminal pro-peptide. Active cathepsin S (Cat S) is co-localized with integrin 3 as a receptor on the surface of vascular smooth muscle cells (SMC), playing an important role in SMC-mediated extracellular matrix (ECM) degradation. It has been evidenced that cathepsins are implicated in arterial vascular diseases through their activation, liberation, and modification of angiogenic growth factors, cytokines, and proteases associated with degradation of lipid metabolism, cell events (migration, invasion, proliferation, and apoptosis), angiogenesis and matrix protein remodeling^5^.

The aim of this study was to assess the association of Cat S with CVD in CKD. We hypothesized that Cat S might increase in earlier stages of CKD and predict the risk of CVD in patients with CKD.

## Subject and Methods

### Study Design and Patient Selection

This was a cross-sectional retrospective study conducted within 1 year at a tertiary center hospital in northern India.

Inclusion criteria were patients with 18 to 65 years of age, with CKD of stage III (eGFR 59-30 mL/min/1.72m^2^), IV (eGFR 29-15 mL/min/1.72m^2^), and V (eGFR < 15 mL/min/1.72m^2^), who were non-dialyzed and who signed an informed consent form. Pregnant or lactating women and patients with malignancy or severe infections were excluded.

### Participants and Groups

**Figure 1:**
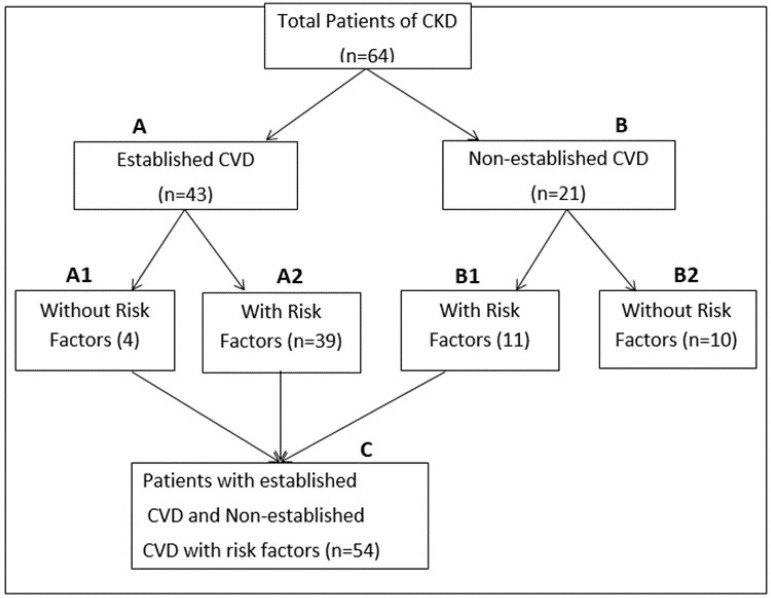
Study design flowchart.

A total of 64 CKD patients were enrolled after their written consent and classified into 2 groups: (A) CKD patients with established CVD and (B) CKD patients with non-established CVD (Figure 1). 2D Echo was done in all patients and the following findings were considered for CVD diagnosis: left ventricular ejection fraction (LVEF) ≤ 55 %, left ventricular hypertrophy, left ventricular diastolic dysfunction, regional wall motion abnormality, mitral regurgitation (MR) / aortic regurgitation / tricuspid regurgitation, stenosis / sclerosis, and left atrial diameter ≥4.9 cm. Patients with a history suggestive of either a cardinal clinical manifestation of CVD/CAD (chest pain, palpitations, breathlessness or syncope) or with a positive finding on 2D Echo or both.

### Physical Examination and Measurements

All patients in the study were subjected to assessment of medical history and thorough clinical examination according to patient records. Routine investigations including complete blood count, random blood sugar, serum electrolytes, urea, creatinine, total protein, total albumin, total calcium, phosphorous, uric acid, vitamin D, parathormone, lipid profile, liver function test, and glycated hemoglobin (HbA1c) were done. The modification of diet in renal disease (MDRD) equation was used for eGFR (mL/min/1.73m^2^) calculation. Specific investigations included measurement of Cat S by ELISA kit and 2D echocardiography of the heart.

### Statistical Analysis

The collected data were computerized and statistically analyzed using Statistical Package for Social Science (SPSS) program version 16.0. Qualitative data are presented as frequencies and relative percentages. The Chi-square test was used to calculate difference between qualitative variables. Quantitative data are presented as mean ± SD (standard deviation). An independent T-test was used to compare differences between quantitative variables in two groups with normally distributed data. The ANOVA F test was used to compare differences between quantitative variables in more than two groups with normally distributed data. A p-value > 0.05 indicated non-significant results, < 0.05 indicated significant results, and < 0.01 indicated highly significant results.

## Results

A total with 64 patients of CKD were enrolled of which 46 (71.88%) CKD patients were male and 18 (28.13%) were female. The majority of patients were in the age group of 41 - 60 years (47%). Forty-four percent of patients was diabetic, hypertension was present in 42.2%, and 39% of patients had diabetic kidney disease. Our results showed that as the stage of CKD increased, there was a significant decrease in hemoglobin and calcium with a significant increase in the level of uric acid, phosphorous, parathormone, and Cat S (Table 1). However, there was no significant association of Cat S with the above biochemical parameters. We looked for independent factors for established CVD in CKD patients, and on multiple sequential regression analysis we found calcium, phosphorous, calcium phosphorous product, high-density lipoprotein (HDL) and low-density lipoprotein (LDL) to be significant. However, Cat S was not found to be an independent risk factor (Table 2). Among CKD patients with established CVD, with non-established CVD with risk factors (54, group C), and with non-established CVD without risk factors (10, group B2) (Figure 1) age, random blood sugar, HbA1C and Cat S were statistically significant (Table 3). At the cut off value of 6.8 for Cat S, the odds ratio for CVD was significant for CKD with established CVD (n=43, group A) compared with CKD with non-established CVD (n=21, group B) (p-value was 0.03). After comparison of all pooled patients of CKD with established CVD as well as patient with risk factors for CVD (n=54, group C) vs. non-established CVD without risk factors (n=10, group B2), the odds ratio for CVD further increased (p-value = 0.006) (Figure 1 and Table 4).

**Table 1:** Biochemical parameters with different stages of CKD

Variables	Stage 3		Stage 4		Stage 5		p-Value
	Mean	±SD	Mean	±SD	Mean	±SD	
Hb (gm/dl)	12.69	1.93	9.87	2.27	9.18	1.95	<0.001*
RBS (mg/dl)	128.60	39.61	139.45	38.42	130.88	56.67	0.737
Urea (mg/dl)	58.50	58.26	72.45	25.23	138.33	45.48	<0.001*
Creatinine (mg/dl)	1.76	0.39	2.52	0.56	6.51	2.70	<0.001*
e-GFR (ml/min/1.73m^2^)	42.55	9.19	24.05	2.82	10.08	3.72	<0.001*
Total Protein (gm/dl)	7.03	0.87	6.56	1.18	6.63	1.23	0.369
Total Albumin (gm/dl)	3.99	0.60	3.77	0.61	3.68	0.89	0.348
Calcium (mg/dl)	8.70	0.68	8.43	1.03	8.10	0.96	0.098
Phosphorous (mg/dl)	4.45	1.88	5.25	1.65	5.80	2.39	0.094
Ca X PO_4_ (mg/dl)	38.36	14.65	44.70	16.84	16.84	18.16	0.256
Uric acid (mg/dl)	7.09	2.19	7.13	2.01	8.94	3.11	0.025*
Vitamin D (ng/ml)	27.51	17.70	20.67	9.50	18.53	23.63	0.258
i-PTH (pg/ml)	87.60	45.49	89.15	60.96	219.29	190.77	0.001*
Cholesterol (mg/dl)	152.20	44.12	159.40	37.68	148.96	43.10	0.707
Triglycerides (mg/dl)	157.90	78.53	163.80	79.42	126.50	58.81	0.184
HDL (mg/dl)	55.60	34.00	47.75	11.50	50.17	24.92	0.602
VLDL (mg/dl)	45.20	20.42	47.20	28.35	34.63	18.84	0.145
LDL (mg/dl)	52.30	26.81	50.40	29.30	62.75	36.47	0.374
HbA1C (%)	6.38	1.32	6.80	2.23	5.76	1.38	0.124
Cathepsin S (ng/ml)	7.8	3.9	8.2	2.9	10.57	3.77	0.028*

Hb: Hemoglobin, RBS: Random Blood Sugar, e GFR: estimated Glomerular Filtration Rate, Ca X PO_4_: calcium phosphorous product, iPTH: intact Parathormone, HDL: high-density lipoprotein, LDL: low-density lipoprotein, VLDL: very low-density lipoprotein, HbA1C: Glycated hemoglobin. A p value < 0.05 is significant.

**Table 2:** Binary multivariate logistic regression (forward-wald) analysis of CKD with established cvd group (43) to determine the independent risk factors for cvd in CKD patients

	B	Std. Error	Wald	p-Value	Exp (B)	95% CI	
						Lower Bound	Upper Bound
Hb (gm/dl)	0.04	0.36	0.01	0.907	1.04	0.52	2.10
RBS (mg/dl)	-0.01	0.02	0.27	0.605	0.99	0.95	1.03
Urea (mg/dl)	-0.02	0.03	0.22	0.641	0.99	0.93	1.05
Creatinine (mg/dl)	-0.48	0.49	0.94	0.331	0.62	0.24	1.62
e-GFR (ml/min/1.73m^2^)	-0.08	0.08	0.85	0.357	0.93	0.79	1.09
Protein (gm/dl)	-0.67	1.07	0.39	0.531	0.51	0.06	4.18
Albumin (gm/dl)	-2.33	1.78	1.71	0.191	0.10	0.00	3.19
Calcium (mg/dl)	-6.34	3.15	4.06	0.044*	0.00	0.00	0.84
Phosphorous (mg/dl)	-10.48	5.08	4.26	0.039*	0.00	0.00	0.59
Ca x PO_4_ (mg/dl)	1.32	0.62	4.44	0.035*	3.73	1.10	12.67
Uricacid (mg/dl)	0.52	0.36	2.17	0.141	1.69	0.84	3.39
Vitamin D (ng/ml)	-0.04	0.03	1.31	0.252	0.97	0.91	1.03
i-PTH (pg/ml)	-0.02	0.01	3.86	0.050	0.98	0.96	1.00
Cholesterol (mg/dl)	-0.09	0.06	2.49	0.114	0.92	0.82	1.02
Triglycerides (mg/dl)	-0.01	0.01	1.12	0.291	0.99	0.97	1.01
HDL (mg/dl)	0.15	0.07	5.15	0.023*	1.16	1.02	1.32
VLDL (mg/dl)	0.09	0.06	2.44	0.118	1.10	0.98	1.23
LDL (mg/dl)	0.13	0.06	4.82	0.028*	1.14	1.01	1.28
HbA1C (%)	-0.38	0.63	0.36	0.549	0.69	0.20	2.36
Cathepsin S (ng/ml)	-0.07	0.07	0.88	0.35	0.93	0.81	1.08

Hb: Hemoglobin, RBS: Random Blood Sugar, e GFR: estimated Glomerular Filtration Rate, Ca X PO4: calcium phosphorous product, iPTH: intact Parathormone, HDL: high-density lipoprotein, LDL: low-density lipoprotein, VLDL: very low-density lipoprotein, HbA1C: Glycated hemoglobin. A p value < 0.05 is significant.

**Table 3:** Biochemical investigation of CKD patients with non-established CVD and no risk factors and CKD patients with established CVD and non-established CVD with risk factors

	CKD with non-established CVD and without risk factors (10)		CKD with established CVD and non-established CVD with risk factors (54)		p-Value
	Mean	±SD	Mean	±SD	
Age (years)	34.60	14.27	49.54	10.55	<0.001
Hb (gm/dl)	10.06	3.11	10.57	2.43	0.631
RBS (mg/dl)	103.00	23.36	138.37	47.10	0.001*
Urea (mg/dl)	77.60	31.27	95.61	60.53	0.175
Creatinine (mg/dl)	3.81	2.04	3.77	2.85	0.961
e-GFR (ml/min/1.73m^2^)	21.60	11.13	25.15	15.27	0.398
Protein (gm/dl)	6.78	1.37	6.73	1.08	0.907
Albumin (gm/dl)	3.72	0.87	3.82	0.70	0.729
Calcium (mg/dl)	8.24	1.01	8.41	0.92	0.624
Phosphorous (mg/dl)	5.88	1.34	5.08	2.17	0.140
Ca X PO_4_ (mg/dl)	48.63	12.76	42.44	17.40	0.205
Uric acid (mg/dl)	8.65	1.31	7.64	2.80	0.082
Vitamin D (ng/ml)	17.26	9.63	22.88	19.59	0.177
i-PTH (pg/ml)	111.40	88.28	142.30	145.75	0.378
Cholesterol (mg/dl)	179.20	56.32	148.43	36.70	0.126
Triglycerides (mg/dl)	154.50	90.24	146.76	70.15	0.802
HDL (mg/dl)	65.40	39.75	48.46	20.73	0.218
VLDL (mg/dl)	37.30	18.89	42.70	23.77	0.439
LDL (mg/dl)	73.60	52.92	52.30	25.15	0.241
HbA1C (%)	5.28	0.64	6.46	1.78	0.001*
Cathepsin S (ng/ml)	6.90	3.77	9.38	3.64	0.045*

Data are reported as mean ± SD. Hb: Hemoglobin, RBS: Random Blood Sugar, e GFR: estimated Glomerular Filtration Rate, Ca X PO4: calcium phosphorous product, iPTH: intact Parathormone, HDL: high-density lipid, LDL: low-density lipid, VLDL: very low-density lipid, HbA1C: Glycated hemoglobin, p value < 0.05 is significant.

**Table 4:** Comparison of cathepsin s in different groups

Cathepsin S level (ng/ml)	CKD with established CVD (43)		CKD with non-established CVD (21)		Odds ratio	^1^p-Value
≥ 6.8	n	%	n	%		
	34	79.0	11	52.3	3.4	0.03
< 6.8	9	21.0	10	47.7		
Cathepsin S level (ng/ml)	CKD with established CVD and non-established CVD with risk factors (54)		CKD with non-established CVD and without risk factors (10)		Odds Ratio	^1^p-Value
	n	%	n	%		
≥6.8	42	77.8	3	30.00	8.1	0.006*
<6.8	12	22.2	7	70.00		

A p value < 0.05 is significant. CKD: chronic kidney disease; CVD: cardiovascular disease.

According to stage, CKD patients with established CVD were 13 (65%), 14,(70%), and 16,(66.6%) in stages 3, 4, and 5, respectively.

A cut off of 6.8 for serum Cat S showed the probability of CVD to be 55%, 70% and 83% in stages 3, 4 and 5 respectively. Hence Cat S, can be used as an early biomarker for CVD in patients with CKD.

## Discussion

The high incidence of cardiovascular events in CKD warrants an accurate evaluation of risk aimed at reducing the burden of disease and its consequences. Several biomarkers have been used to identify patients at high risk in the general population.

Chronic vascular inflammation combined with the imbalance in mineral bone disorder due to calcium phosphate metabolism increases the risk for cardiovascular disease due to accelerated atherosclerosis and arteriosclerosis. Cat S plays a crucial role in various conditions that involve large biological systems, such as autoimmune disease, cardiomyopathy, heart valve disease, and atherosclerosis. Among various cathepsins, such as cathepsin B, C, F, H, K, L, O, S, V, W, and Z, Cat S is thought to generate bioactive elastin peptides due to potent cysteine protease, which cleaves elastin and leads to the promotion of cardiovascular inflammation and calcification^4^.

Here we evaluated Cat S as a biomarker in CKD patients. We found that Cat S increased as the eGFR declined and the stage of CKD advanced. It was found that as the glomerular filtration rate decline, Cat S and markers of inflammation-related endothelial dysfunction increased and hence Cat S is suggestive of inflammatory-related endothelial dysfunction for prediction of cardiovascular morbidity and mortality. This indicated that Cat S activity increases with CKD progression, suggesting that Cat S may be a therapeutic target to prevent cardiovascular complications in CKD^4^^,^ 6.

In our study, the overall prevalence of CVD among 64 CKD patients was 67.19% (n=43), which was also found in other studies. It was also found that CKD is an independent risk factor for CVD and the majority of patients die due to CVD rather than due to progress to end-stage renal disease (ESRD)^7^.

Diabetes mellitus and hypertension were major risk factors for CVD in CKD, which is in accordance with other studies^8^^,^ 9 ^-^ 10. However, diabetes mellitus was significantly associated with CVD in the CKD group.

As age advanced, the risk for diabetes mellitus and its complications, such as atherosclerosis, also increased. In this subgroup, Cat S was significantly associated with CVD. The increase in serum Cat S level in atheroma patients was more significant than in non-involved patients^11^.

In this study, we found calcium, phosphorous, calcium phosphorous product, HDL, and LDL to be significant factors for CVD in patients with CKD^12^^-^ 17. HDL particles are highly heterogeneous and under pathological conditions, including CKD, HDL properties may be altered, resulting in increased cardiovascular risk^12^.

Cat S in cardiovascular calcification is associated with mineral imbalance found in diabetes and CKD. While looking at the role of fibroblasts in medial vascular calcification, it was found that calcified nodules are formed in the presence of elastin degradation products and transforming growth factor beta 1 (TGF-b1). It has been shown that elastin degradation peptides can induce calcification of mesenchymal cells in vitro. These findings suggest that elastin degradation could induce calcification of vascular SMCs and valvular myofibroblasts and thus mediate calcification^7^. Also, the release of Cat S by activated macrophages within the vascular wall degrades the elastic fibers of the tunica elastica, a process that contributes to vascular wall degeneration, media calcification, and aneurysm formation^4^^,^ 18.

In our study, using a cut-off value of 6.8 for Cat S, a higher CVD risk was found when compared with CKD patients with and without established CVD (p value=0.03, odds ratio=3.4). It has been shown that Cat S inhibition reduces the progression of atherosclerotic lesions, hence reducing risk of CVD.^19^ Furthermore, in an experimental mouse model of systemic lupus erythematosus and lupus nephritis, Cat S inhibition by the RO5444101 inhibitor showed therapeutic benefits with a significant decrease in Cat S, elastin degradation, calcification, and plaque size^2^^,^ 20.

In our study, the values of Cat S, age, HBA1c, and RBS were found to be significantly increased in patients with CKD with established CVD and with risk factors. This is in agreement with another study^11^. Also we found that a cut-off value of 6.8 for Cat S was significant in this group (p-value 0.006, OR: 8.1).

Circulating Cat S levels and mortality were correlated, as were Cat S activity and the development of CVD due to increase in the formation and destabilization of atherosclerotic plaques. This indicates that Cat S activity increases with CKD progression, suggesting that inhibition of Cat S may be a therapeutic target to prevent cardiovascular complications in CKD^9^^,^ 21.

## Conclusion

Diabetes mellitus and hypertension are major risk factors for CKD and CVD. Cat S increased as the eGFR declined and the stage of CKD advanced. In CKD patients with CVD and with risk factors such as age, HBA1C was found to be significantly increased with higher Cat S levels. Cat S showed a significant association with CVD and may be used as an early biomarker for CVD in CKD as evidenced by the increased level of Cat S in early stage CKD. Large multicentre prospective studies are needed to validate the findings of the present study.
